# Achieved Weight Gain and Return to Work After Intervention With Traditional Japanese Acupuncture for Avoidant Restrictive Food Intake Disorder: A Case Report

**DOI:** 10.7759/cureus.61912

**Published:** 2024-06-07

**Authors:** Takuya Masuda, Yu Takeshita, Koichiro Tanaka

**Affiliations:** 1 Division of General Internal Medicine and Rheumatology, Mitsui Memorial Hospital, Tokyo, JPN; 2 Department of Traditional Medicine, Toho University, Tokyo, JPN; 3 Department of Western Medicine, Hokushin-kai, Academic Society of Traditional Japanese Acupuncture and Moxibustion, Osaka, JPN; 4 Department of Integrative/Complementary Medicine, Acupuncture Clinic, Seimei-in, Tokyo, JPN; 5 Department of Oriental Medicine, Hokushin-kai, Academic Society of Traditional Japanese Acupuncture and Moxibustion, Osaka, JPN

**Keywords:** dashin of mubun-ryu style, eating disorder, arfid, traditional japanese acupuncture and moxibustion, oriental medicine

## Abstract

Avoidant restrictive food intake disorder (ARFID) is newly established as a category of eating disorder (ED). Acupuncture is one treatment option for ED. However, no cases of acupuncture treatment of ARFID have been reported.

A 28-year-old female presented with reduced food intake and weight, abdominal bloating, abnormal sense of taste, and tongue pain. Her body weight (BW) had been around 50 kg until four years previously. Three years before, her symptoms occurred, and her BW decreased to 36.5 kg after experiencing excessive mental stress at her workplace. She was diagnosed with ARFID by a psychosomatic physician and tended to refuse her prescribed antipsychotic drugs. She was treated weekly with the Hokushin-kai style, a traditional Japanese acupuncture, and the moxibustion method. After one month, the patient felt somewhat better and returned to work once a week for the first time in two years. Four months later, her BW started to increase. After 10 months, her BW had increased to 48 kg. Her acupuncture treatment continues.

This case suggests acupuncture as an optional treatment for ARFID. Further studies, such as a combination of medications and acupuncture, would be desirable.

## Introduction

Avoidant restrictive food intake disorder (ARFID) is newly established as a category of eating disorder (ED) in the Diagnostic and Statistical Manual of Mental Disorders (DSM-5) [1,2]. The prevalence of ARFID is estimated at 14.6% among ED patients in Japan [3]. ARFID is distinguished from anorexia nervosa (AN) and bulimia nervosa (BN) by its absence of body shape or weight concerns. The characteristics of ARFID are (1) avoidance based on sensory characteristics of food, (2) concern about aversive consequences related to eating, and (3) apparent uninterest in eating or food [1]. Treatment for ARFID includes medications, psycho-behavioral therapy, and cognitive behavioral therapy [1,2]. There is a lack of research into its pathology, and evidence for therapeutic and pharmacological interventions for ARFID is limited [2,4].

Acupuncture is one treatment option for EDs [5-7]. Needles are inserted into specific parts of the body, termed acupoints. A randomized crossover study [6] revealed that acupuncture for nine ED patients significantly improved their quality of life and decreased their anxiety and perfectionism. A practice guideline of acupuncture for AN has been published [7].

The treatment includes various methods, such as traditional Chinese medicine (TCM), Western medicine, and the Japanese method. However, no cases of acupuncture treatment by the Japanese method for ED or ARFID have been reported in English.

Here, we show a case of ARFID treated with the Hokushin-kai style [8], a traditional Japanese acupuncture, and moxibustion.

## Case presentation

A 28-year-old female was presented to an acupuncture department in our hospital because of her reduced food intake and weight, abdominal bloating, constipation, insomnia, abnormal sense of taste, and tongue pain. She had been in good health, with a body weight (BW) of around 50 kg, until four years before this consultation. Three years before, she was suffering excessive mental stress at her workplace.

At the same time, she started to feel her sense of taste strengthen to saltiness and sweetness. Her BW decreased to 36.5 kg (body mass index {BMI}: 14.5) for fear of abdominal bloating after eating, despite having an appetite. She began a leave of absence from her job. No self-induced vomiting nor inappropriate use of diuretics and laxatives was noted.

Two years before, she came to our hospital and was diagnosed with ARFID by a psychosomatic physician. She had no past medical history. Laboratory tests revealed no evidence of disease such as thyroid or zinc deficiency. She tended to refuse her prescribed antipsychotic drugs, due to adverse effects of nausea or amnesia in the past. Kampo medicine such as Hangekobokuto or Tokishakuyakusan was prescribed, as well as 10 mg of amitriptyline, but her BW varied around 31-36 kg. Acupuncture treatment was started.

**Figure 1:**
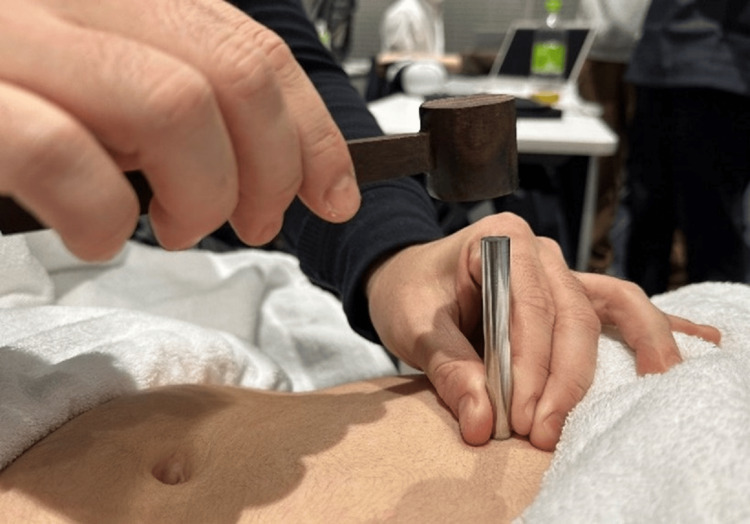
The procedure of the Dashin method. In Mubun-ryu, the abnormality of mental and bodily status is expressed as various physical findings (e.g., tension, tenderness, heat, coldness, flaccidity, and perspiration) on particular surface areas of the abdomen. Tapping the needle at specific positions, such as those with tension or tenderness, on the abdominal surface. Image credits: Yu Takeshita.

From the perspective of Oriental medicine, we treated with Hokushin-kai style [8], a traditional Japanese acupuncture method, and moxibustion. Treatment sessions were held weekly. One or two sterilized disposable needles (I’SSIN Co., Hyogo, Japan) were inserted into each acupoint for 10 minutes, to a depth of 4-10 mm, with no manipulations. We added as needed a technique involving a tapping needle on the abdomen, termed Dashin (tapping an acupuncture needle with a wooden hammer on the surface of the abdomen) (Figure 1) [8]. Acupoints, needle size, needling method, and the use of Dashin at each session of acupuncture treatment are shown in Table 1.

**Table 1:** Acupoints, needle size, needling method, and added Dashin of each acupuncture treatment session over 10 months. R, right; L, left; D, draining; T, tonifying method

Number of treatments	Acupoint 1	Needle size (mm)	Needling method	Acupoint 2	Needle size (mm)	Needling method	Retention time (minute)	Dashin
1	L, Pishu (BL20)	0.2 × 20	D	L, Zhaohai (KI6)	0.18 × 10	T	10	No
2	L, Weishu (BL21)	0.2 × 40	D	-	-	-	10	Yes
3	R, Pishu (BL20)	0.2 × 20	T	-	-	-	10	No
4	L, Tianshu (ST25)	0.2 × 40	D	-	-	-	10	No
5	L, Huaroumen (ST24)	0.2 × 20	D	L, Sanyinjiao (SP6)	0.2 × 20	D and T	10	Yes
6	L, Weishu (BL21)	0.2 × 20	D	-	-	-	10	Yes
7	L, Weishu (BL21)	0.2 × 40	D	-	-	-	10	Yes
8	L, Weishu (BL21)	0.2 × 40	D	-	-	-	10	Yes
9	L, Pishu (BL20)	0.2 × 40	D	-	-	-	10	Yes
10	L, He Gu (LI4)	0.2 × 20	D	R, Sanyinjiao (SP6)	0.18 × 10	T	10	Yes
11	L, Huaroumen (ST24)	0.2 × 40	D	R, Gongsun (SP4)	0.2 × 20	T	10	Yes
12	L, Weishu (BL21)	0.2 × 40	D	L, Zhaohai (KI6)	0.18 × 10	T	10	Yes
13	L, Baihui (GV12)	0.2 × 20	D	L, Zhaohai (KI6)	0.18 × 10	T	10	Yes
14	R, Neiguan (PC6)	0.18 × 10	D	R, Zhaohai (KI6)	0.18 × 10	T	10	Yes
15	L, He Gu (LI4)	0.2 × 20	D	L, Sanyinjiao (SP6)	0.2 × 20	D and T	10	Yes
16	L, Danshu (BL19)	0.20 × 40	D	L, Zhaohai (KI6)	0.18 × 10	T	10	Yes
17	L, Baihui (GV12)	0.2 × 20	D	L, Sanyinjiao (SP6)	0.2 × 20	D and T	10	Yes
18	L, Shenmen (HT7)	0.18 × 10	T	L, Sanyinjiao (SP6)	0.2 × 20	D and T	10	Yes
19	L, Houxi (SI3)	0.18 × 10	D	L, Gongsun (SP4)	0.2 × 20	D and T	10	Yes
20	L, Burong (ST19)	0.2 × 20	D	L, Zhaohai (KI6)	0.18 × 10	T	10	No
21	R, Chengman (ST20)	0.2 × 20	D	L, Sanyinjiao (SP6)	0.2 × 20	D and T	10	Yes
22	L, Xinshu (BL 15)	0.2 × 20	T	R, Zhaohai (KI6)	0.18 × 10	T	10	Yes
23	L, Burong (ST19)	0.2 × 20	D	R, Zusanli (ST36)	0.2 × 20	T	10	Yes
24	R, Houxi (SI3)	0.18 × 10	D	L, Fenglong (ST40)	0.20 × 40	D	10	Yes
25	L, Tianshu (ST25)	0.20 × 40	D	R, Shinmon (HT7)	0.18 × 10	T	10	No
26	R, Burong (ST19)	0.2 × 20	D	L, Gongsun (SP4)	0.2 × 20	T	10	No
27	R, Sanjiaoshu (BL22)	0.20 × 40	T	-	-	-	10	Yes
28	L, Tianshu (ST25)	0.2 × 20	D	-	-	-	10	Yes
29	R, Fenglong (ST40)	0.25 × 48	D	-	-	-	10	Yes

After the procedure, the patient’s abdominal bloating and insomnia improved for about five days. Sometimes, she did not attend a treatment session, and her abdominal bloating and insomnia were worse about one week after the session of acupuncture. No change was noted to her abnormal sense of taste or tongue pain after the treatment.

One month after commencing the treatment, she felt less unwell and tired and returned to her work once a week for the first time in two years. From three to four months after the first acupuncture treatment, she was prescribed 1 mg of risperidone, 25 mg of quetiapine, and 1 mg of aripiprazole. However, the use of these drugs was stopped due to her hypersomnia.

**Figure 2:**
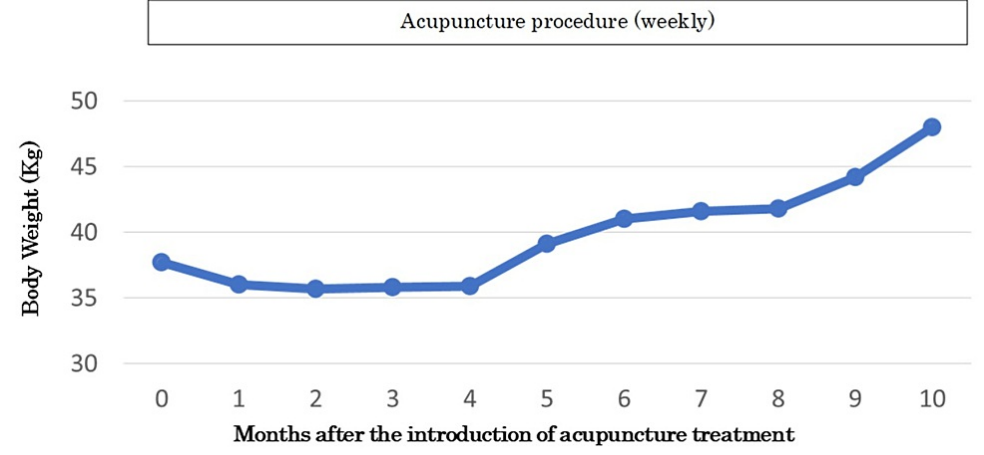
Therapeutic course of acupuncture treatment. Treatment sessions were held weekly. One month after the treatment, the patient returned to her work, once a week. From three to four months after the first treatment of acupuncture, she was prescribed 1 mg of risperidone, 25 mg of quetiapine, and 1 mg of aripiprazole. The use of these drugs was stopped due to her hypersomnia. Four months later, her BW started to increase. After 10 months, her BW had increased to 48 kg. BW: body weight

Four months later, the patient was consuming a stable amount of food. Her BW gradually increased. She went to work four days a week. After 10 months, her BW had increased to 48 kg (Figure 2). Her abnormal sense of taste, tongue pain, insomnia, and abdominal bloating remain. The acupuncture treatment continues. Acupuncture was performed by a clinical physician (three years of experience in acupuncture), and no adverse events occurred.

## Discussion

This is the first English-language case report of acupuncture treatment with the Japanese method for ED, especially ARFID. Weight gain and a return to work were achieved after the intervention of traditional Japanese acupuncture once a week. As mentioned above, acupuncture for nine ED patients significantly improved their quality of life and decreased their anxiety and perfectionism [6]. However, the participants were AN or BN patients, not ARFID patients.

World Federation of Societies of Biological Psychiatry (WFSBP) guidelines [2] show that evidence for therapeutic and pharmacological interventions for ARFID is limited. Some cases reported the effectiveness of antidepressants and antipsychotics such as mirtazapine and olanzapine on ARFID [2].

Various studies reveal the altered processing of a reward system in AN or BN [4,9], and a functional neuroimaging study of the altered processing of rewarding and aversive food stimuli in acute and recovered AN [10,11] is an example. ARFID is similar to AN and assumes the pathogenesis of the altered processing of a reward system. Interestingly, a functional MRI study [12] shows that acupuncture regulates the signal limbic system.

In our case, the patient was prescribed risperidone, quetiapine, and aripiprazole from three to four months after the first treatment of acupuncture. Interestingly, her BW started to increase one month after the prescription, despite its withdrawal. Acupuncture plays an antidepressant role, regulates neurotransmitter systems and neuroendocrine axis, improves neuroplasticity, and has anti-inflammation effects [13]. There might be some synergistic effect between acupuncture therapy and such drugs.

Acupuncture was first developed around 100 BC. In Oriental medicine, the evaluation and treatment of disharmony between mental and bodily status is a specialty. Acupuncture is now used around the world for various mental symptoms. For instance, an American Society of Clinical Oncology (ASCO) guideline [14] recommends acupuncture for treating symptoms of anxiety.

Chinese medicine, including acupuncture, was introduced into Japan in the Asuka to Nara era (AD 592-794) [15]. Dashin of Mubun-ryu style was established by Isai Misono in the Muromachiera (AD 1336-1573) in Japan [8,16-19]. In Mubun-ryu, the abnormality of mental and bodily status is expressed as various physical findings on particular surface areas of the abdomen (Figure 2). On physical examination, these findings are recognized as tension, tenderness, heat, coldness, flaccidity, and perspiration. In this patient, tension existed in the heart, spleen, stomach, liver, and large intestine. These findings suggested that our patient had abnormalities of mental status, emotional stress, intestinal immobility, and digestion system.

Taking the findings into account, we diagnosed her as having a “liver-stomach disharmony pattern,” “dampness-heat,” and “heart blood deficiency pattern.” In the theory of Oriental medicine, the function and condition of the mind and body are divided into five viscera and six bowels, termed the Zang Fu theory of TCM. In this patient, chronic mental stress caused “liver” (dominating the emotional side) Qi stagnation and functional impairment of the “stomach” (liver-stomach disharmony pattern). Each problem produced “heat” and “dampness” (dampness-heat). “Heat” was finally impairing the “heart” (dominating the consciousness, mental side) (heart blood deficiency pattern). Patients with “heart” abnormalities often exhibit sleeping disorders and tongue pain. The “dampness-heat” abnormalities are abdominal bloating and an abnormal sense of taste.

**Figure 3:**
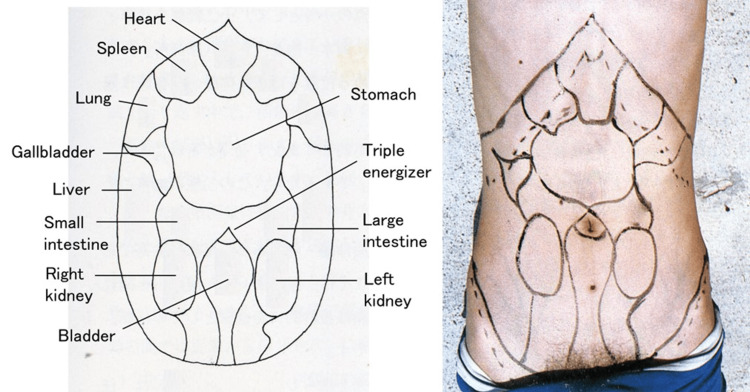
Mubun-ryu abdominal illustration of the Zang Fu theory (TCM) of five viscera and six bowels. The abnormality of mental and bodily status (Zang Fu) expresses various findings on particular surface areas of the abdomen. These findings are recognized as tension, tenderness, heat, coldness, flaccidity, and perspiration. Image credits: Yu Takeshita. TCM: traditional Chinese medicine

In treatment, we tapped the needle with a wooden hammer for Dashin on those same areas of the surface of the abdomen for the therapeutic purpose of stabilizing the mind and promoting intestinal motility (Figure 3). After the tapping, the tension on the abdominal surface was lowered. The reason for using Dashin is as follows: it is known from extensive clinical experience that if a patient is of weak status, it is safer to use Dashin as it imposes no burden on a patient’s mental and physical status, compared to inserting an acupuncture needle. Actually, mental vulnerability was expected in our patient, and we inserted only one or two needles, with great care, so that undesired effects such as serious malaise might not be seen.

In the past, Japan’s government attempted to abolish the practice of acupuncture in favor of Western medicine, despite acupuncture’s excellent clinical effects during the Meiji era (AD 1868-1912) [15]. At present, no system of collaboration has been established between doctors and acupuncturists for the provision of treatment to patients in Japan [20]. Further studies and collaboration between doctors and acupuncturists will be needed for the treatment of ED patients.

## Conclusions

We present a case of achieved weight gain and a return to work after the intervention of traditional Japanese acupuncture for ARFID. No previous study revealed that acupuncture treatment was effective in improving weight for ED. This case suggests that the traditional Japanese acupuncture method would be an optional treatment for ARFID. Further studies, such as on traditional Japanese acupuncture that was effective for other types of EDs, as well as ARFID, would be desirable.
